# Poisons

**Published:** 1879-08

**Authors:** E. H. Gibbs


### POISONS.

By E. H. Gibbs, M. D.

The first thing to be done when a person has swallowed laudanum, arsenic, or other poisonous thing, is to take an emetic in order to empty the stomach as soon as possible and prevent the poison from being absorbed and taken into the blood. For this purpose, stir a tablespoonful of *mustard* in a tumbler of cold or warm water, and let the person poisoned drink slowly until it produces free vomiting. A tablespoonful or more of common salt, in the same quantity of water, will answer the purpose, but not quite as well If vomiting does not take place within a minute or two, let the person tickle the back part of the throat with his finger. Many persons can induce free vomiting by this means alone. When vomiting ceases swallow the white of one or two eggs for the purpose of neutralizing the effect of any small portion of the poison which may be left in the stomach after vomiting. If eggs are not at hand use butter, oil, lard, cream, or milk. A physician should be sent for in all cases. The directions above given are intended to be only temporary expedients to save life. Appropriate medical treatment is frequently necessary to prevent unfortunate results from the various poisons.

